# Optimizing the effective spot size and the dosimetric leaf gap of the AcurosXB algorithm for VMAT treatment planning

**DOI:** 10.1002/acm2.13256

**Published:** 2021-05-27

**Authors:** V. Passal, M. Barreau, T. Tiplica, S. Dufreneix

**Affiliations:** ^1^ Institut de Cancérologie de l’Ouest Angers France; ^2^ LARIS Systems Engineering Research Laboratory University of Angers Angers France

**Keywords:** AcurosXB, beam model, design of experiment, dosimetric leaf gap, volumetric modulated arc therapy

## Abstract

**Purpose:**

The aim of this study is to provide and test a new methodology to adjust the AcurosXB beam model for VMAT treatment plans.

**Method:**

The effective target spot size of the AcurosXB v15 algorithm was adjusted in order to minimize the difference between calculated and measured penumbras. The dosimetric leaf gap (DLG) was adjusted using the asynchronous oscillating sweeping gap tests defined in the literature and the MLC transmission was measured. The impact of the four parameters on the small field output factors was assessed using a design of experiment methodology. Patient quality controls were performed for the three beam models investigated including two energies and two MLC models.

**Results:**

Effective target spot sizes differed from the manufacturer recommendations and strongly depended on the MLC model considered. DLG values ranged from 0.7 to 2.3 mm and were found to be larger than the ones based on the sweeping gap tests. All parameters were found to significantly influence the calculated output factors, especially for the 0.5 cm × 0.5 cm field size. Interactions were also identified for fields smaller than 2 cm × 2 cm, suggesting that adjusting the parameters on the small field output factors should be done with caution. All patient quality controls passed the universal action limit of 90%.

**Conclusion:**

The methodology provided is simple to implement in clinical practice. It was validated for three beam models covering a large variety of treatment types and localizations.

## 1 INTRODUCTION

Although modulated treatment plans are commonly delivered in clinical practice, the modelization of the multileaf collimator (MLC) in the treatment planning systems (TPS) is known to be delicate and can affect dose calculation accuracy.^1^ The Eclipse TPS (Varian Medical Systems, Palo Alto) models the rounded leaf end of the MLC based on two parameters: the MLC transmission (T) and the dosimetric leaf gap (DLG). Two other parameters can be tuned by the user to adjust the field output factors of small fields and the penumbras of dose profiles: the effective target spot size in the X and Y directions. As already shown in the literature, the effective target spot size can also affect the dosimetric accuracy of modulated plans.^2^, ^3^

All four parameters are associated with recommendations provided by the manufacturer^4^: measurement conditions for the transmission are provided, values for the effective target spot size are given depending on the algorithm and it is suggested to adjust the DLG based on the sweeping gap method. These recommendations have widely been discussed in the literature.^3^, ^5^, ^6^, ^7^, ^8^, ^9^ For example, Gardner et al.^3^ showed that for intracranial SRS VMAT planning on an Edge accelerator, the 0.5 mm effective target spot size yielded highest passing rates compared to the vendor recommended 1.0 mm effective target spot size. Because the sweeping gap tests do not account for the tongue and groove effect,^5^ some authors suggested adjusting the DLG in order to improve the patient quality controls^7^, ^8^, ^9^ which resulted in an increase of the DLG value compared to the sweeping gap tests. For example, the adjusted DLG value reported by Kim et al.^7^ was 0.9 mm for a 6 MV FFF beam on an Edge accelerator whereas the physical DLG based on sweeping‐gap measurement was only 0.27 mm. This methodology based on patient quality controls, however, results in a beam model dedicated to a treatment type and localization which can be penalizing in clinical practice when different treatments plans are generated using the same beam model. Illustrating the complexity of the DLG determination, the survey conducted by Glenn et al.^10^ of self‐reported TPS beam modeling parameter values revealed a large variability for the DLG values for the AAA and AXB algorithms.

The aim of this study is to provide and test a methodology to adjust the AcurosXB beam model for VMAT treatment plans. The effective target spot size were adjusted in order to match the measured penumbras and the DLG was determined based on the aOSG tests defined by Hernandez et al.^5^ The impact of the beam parameters on small field output factors was investigated using a design of experiment methodology. The methodology suggested was tested and validated for three beam models encompassing two energies and two MLCs. A comparison with the manufacturer recommendations was also performed.

## 2 MATERIALS AND METHODS

Three beam models were considered in this study: one 6 MV beam on a TrueBeam Tx associated with a 120 Millennium MLC, one 6 MV beam on a TrueBeam STx associated with a 120 High Definition (120 HD) MLC, and one 6 MV FFF beam on a TrueBeam STx associated with a 120 HD MLC.

All calculations were performed with AcurosXB v15 algorithm with a 1 mm calculation grid. During commissioning, percentage depth dose, profiles in the crossline direction, diagonal profiles and field output factors were measured for field sizes ranging from 2 cm x 2 cm to 40 cm x 40 cm with a CC13 (IBA) ionization chamber. Although, as stated by the manufacturer, “beam model should be accurate even though the measurement data does not contain very small field sizes (1 × 1 cm^2^ and 2 × 2 cm^2^)”,^4^ data for the 2 cm x 2 cm field were measured as recommended by the guidelines from the AAPM.^11^ No data was measured during commissioning for the 1 cm x 1 cm field since “depth dose curve and profile measurements for field sizes smaller than 2 × 2 cm^2^ are ignored by the configuration program”.^4^ Correction factors of the IAEA/AAPM TRS 483^12^ were applied to define the field output factors.

### 2.1 Adjustment of the source sizes on the penumbra

The effective target spot size in the X and Y directions (respectively σ_X_ and σ_Y_) models the broadening of the penumbra in X and Y direction. The modeling is done by applying a Gaussian smoothing to the energy fluence of primary photons. This parameter equals the width of the Gaussian distribution in the X/crossline or Y/inline direction at isocenter plane, expressed in millimeters.^4^

σ_X_ and σ_Y_ were adjusted by comparing calculated and measured penumbras. Measurements were conducted with a Razor diode (IBA) with a sensitive area of 0.6 mm diameter at 10 cm depth with a Source‐Surface Distance (SSD) of 90 cm and a measurement step of 0.1 mm in penumbra region (0.2 mm elsewhere). Similarly to the literature,^13^ five field sizes defined by the MLC were studied: 0.5 cm x 0.5 cm and from 1 cm x 1 cm to 4 cm x 4 cm with a 1 cm stepping. Jaws were set to 10 cm x 10 cm. Penumbras were defined as the distance between the 20% and the 80% dose levels with the 100% set at the beam central axis for each profile, even for the FFF beam with regard to the small field sizes studied. σ_X_ and σ_Y_ were individually incremented from 0 to 2 with a 0.2 mm stepping. The mean deviation between calculated and measured right and left penumbras for all five field size was reported in the crossline (σ_X_) and inline (σ_Y_) directions.

### 2.2 Determination of the DLG using the aOSG tests

The dosimetric leaf gap accounts for dose transmission through the rounded MLC leaves. The exact value of the parameter depends on the MLC device and the energy spectrum of the accelerator. Hernadez et al.^5^ provided comprehensible procedures for the commissioning of TPSs regarding the tongue‐and‐groove effect. They are based on asynchronous oscillating sweeping gap tests (a‐OSG) where a uniform MLC gap repeatedly moves across the field at a constant speed during a full gantry rotation. Contrary with sweeping gap tests, the tongue‐and‐groove effect is incorporated by introducing a shift between the positions of adjacent leaf pairs. For each gap width *g*, a range of shifts *s* were evaluated and the tongue‐and‐groove fraction was expressed as TG fraction = *s*/*g*. Three gaps (10, 20, and 30 mm) and five TG fractions (0, 0.25, 0.5, 0.75, and 1) were investigated corresponding to a large variety of complexity for patient treatment plans. Measurements were conducted with a FC65 (IBA) ionization chamber positioned at the center of a cylindrical homogeneous phantom. The DLG parameter was incremented from 0 to 3.4 mm. The mean absolute difference between calculated and measured doses for the fifteen plans was reported. The DLG was also measured using the commonly sweeping gap test for comparison purpose.

The leaf transmission factor can be estimated as the ratio of the measured dose in an open field and the measured dose when using the same field size with all MLC leaves closed behind the jaws. Measurements were performed with a PPC40 ionization chamber at 10 cm depth with a source‐to‐surface distance of 90 cm. For 120 HD MLC the field size was set to 10 cm x 15 cm, and for 120 Millennium MLC it was set to 10 cm x 25 cm as recommended by the manufacturer.^4^

### 2.3 Influence of the parameters on small output factors using a design of experiments methodology

The effective target spot size, the DLG and the transmission are likely to impact small field output factors.^13^ Moreover, interactions between these four parameters may exist, i.e., the influence of one parameter on small field output factors may depend on the value of another parameter. The design of experiment methodology is an optimization technique that efficiently reveals the influence of some inputs (referred to as factors) on outputs of interest.^14^ More specifically, Taguchi arrays which are used in the construction of the experimental plan, allow independent estimation of the factors’ impact on the outputs and also the magnitude of their interaction.^15^ The choice of an orthogonal array is made with regard to the number of factors and interactions to be evaluated and also of the number of levels considered for each factor. For the present study, four factors were investigated (σ_X_, σ_Y,_ DLG and transmission) with three levels each in order to take into account any possible nonlinearity between the output of the array and the factors’ levels. A Taguchi L27 design of experiment was chosen. The description of the 27 trials are described in supplementary data. The associated levels for each parameter and each beam model are given in Table 1. Values were chosen regarding the results obtained in Sections 3.1 and 3.2.

**Table 1:** Levels of the parameter for each beam model defined for the design of experiment.

	Level	σ_X_ (mm)	σ_Y_ (mm)	DLG (mm)	T (%)
TrueBeam Tx ‐ Millennium MLC ‐ X6	1	0.7	0	0.75	1
	2	1.3	1.1	2.3	1.8
	3	1.8	1.4	3	2
TrueBeam STx ‐ 120 HD MLC ‐ X6	1	0	0	0.2	0.8
	2	0.6	0.6	1.5	1.2
	3	1.2	1.2	2.5	1.8
TrueBeam STx ‐ 120 HD MLC ‐ X6 FFF	1	0	0	0	0.8
	2	0.6	0.6	0.8	1.2
	3	1.2	1.2	1.6	1.8

σX/σY, effective target spot size in the X/Y direction; DLG, dosimetric leaf gap; T, transmission.

Field output factors measurements were performed with a 60019 CVD diamond (PTW) and a Razor diode (IBA) at 10 cm depth with a SSD of 90 cm for field sizes from 0.5 to 2 cm with a 0.5 cm stepping and for field sizes of 3 and 4 cm. Fields were defined by the MLC and jaws were set to 10 cm x 10 cm. A reproducibility smaller than 1% was found between two sets of measurements. Correction factors from the IAEA‐TRS483^12^ and from Casar et al.^16^ were applied to the uncorrected ratio of readings of the 60019 CVD diamond and the Razor diode respectively. The difference between calculated and measured output factors was computed for both detectors and the mean value was considered for the analysis. The factors’ effects were estimated for each level of each factor separately and their significance was interpreted by using an analysis of variance (anova) model with a significance level fixed at α = 1%.

### 2.4 Validation of the beam models

Once the parameters σ_X_, σ_Y,_ DLG, and transmission were determined, the validity of the beam models were checked with patient quality controls. For each beam model, 10 VMAT plans including all localizations commonly treated in clinical practice were optimized and calculated. Associated patient quality controls were performed using EBT3 films placed in a homogeneous cylindrical phantom. A film calibration was performed in a water‐equivalent phantom under reference conditions. Films were scanned 24 h after irradiation using an Epson Expression 10000 XL [US Epson, Long Beach, CA, USA], with transmission mode, 48 bits RGB (16 bits per channel color) and a resolution of 200 dpi (0.35 mm/pixel). The methodology described in Ref. [17] was followed. The 2D measured dose distribution was compared to the calculated one and a 3% ‐ 2 mm global gamma analysis with a 10% threshold was performed in agreement with the AAPM Task Group No. 218.^18^ For comparison purpose, another dosimetry was performed (reoptimization and recalculation) for the same patients based on the beam model following the manufacturer’s recommendations. Associated patient quality controls were performed.

## 3 RESULTS

### 3.1 Measured versus calculated penumbras

**Fig. 1:**
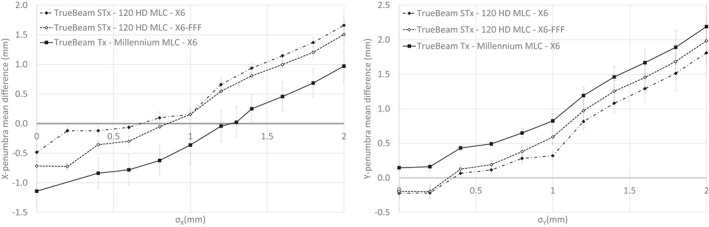
Mean deviation between calculated and measured penumbras in the crossline (σ_X_, left) and inline (σ_Y_, right) directions as a function of the effective target spot size (reference: measurement).

The mean deviation between calculated and measured penumbras for the three beam models studied is shown in Fig. 1 in the crossline (X) and inline (Y) directions as a function of the effective target spot size (σ). Optimal values are given in Table 2. The mean deviation varies approximately linearly with the effective source size. The same trend can be observed for the two energies and two MLCs studied. Except for the Millennium MLC in the inline direction, all curves cross the horizontal axis meaning that there is an effective target spot size value for which calculated and measured penumbras are identical.

**Table 2:** Values for the four parameters of the three beam models investigated in this study.

	σ_X_ (mm)	σ_Y_ (mm)	DLG (mm) *(aOSG test)*	DLG (mm) *(sweeping gap test)*	T (%)
TrueBeam Tx ‐ Millennium MLC ‐ X6	1.25	0	2.3	0.55	1.49
TrueBeam STx ‐ 120 HD MLC ‐ X6	0.7	0.35	1.3	0.38	1.26
TrueBeam STx ‐ 120 HD MLC ‐ X6 FFF	0.8	0.3	0.7	0.24	1.10

### 3.2 Measured versus calculated dose for the aOSG tests

**Fig. 2:**
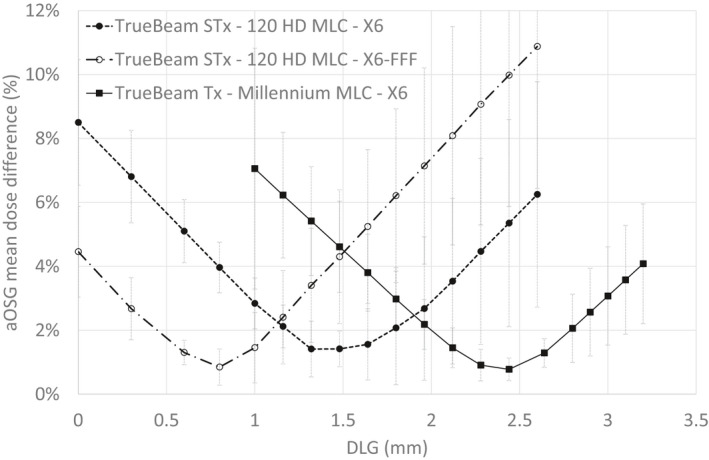
Mean absolute difference between calculated and measured doses for the aOSG tests as a function of the dosimetric leaf gap (DLG).

The mean deviation between calculated and measured doses for the 15 aOSG plans as a function of DLG is plotted in Fig. 2. All curves are approximately parabolic with a minimum between 0.8 and 2.5 mm depending on the MLC and energy considered. All minimums are reached for a mean deviation of approximately 1%. Also interesting to note is that the minimum value is also associated to a smaller standard deviation of the dose differences over the 15 plans, which indicates a better agreement for different plan characteristics.

The measured leaf transmission factor was 1.49% for the 6 MV beam on a TrueBeam Tx associated with a 120 Millennium MLC, 1.26% for the 6 MV beam on a TrueBeam STx associated with a 120 HD MLC, and 1.10% for the 6 MV FFF beam on a TrueBeam STx associated with a 120 HD MLC.

### 3.3 Impact of σ_X_, σ_Y_, DLG, and transmission on the small field output factors

Parameters significantly influencing the field output factors are given in Table 3. A similar trend was observed for all beam models encompassing two energies and two models of MLCs. The number of significant parameters increased with decreasing field size and almost all parameters and interactions were found significant for the 0.5 cm x 0.5 cm field size. No interaction was found significantly above 2 cm x 2 cm. When the DLG or the transmission were considered significant, the dose difference decreased when the DLG/transmission decreased. When σ_X_ and σ_Y_ were considered significant, the dose difference decreased when σX/σY increased.

**Table 3:** Significant parameters on the field output factors (parameters are ordered with decreasing significance).

0.5 cm	1 cm	1.5 cm	2 cm	3 cm	4 cm
σ_Y_	DLG	DLG	DLG	DLG	DLG
DLG	σ_Y_	σ_Y_	T	T	T
σ_X_	σ_X_	σ_X_	σ_Y_		
DLG‐σ_X_ interaction	T	T	σ_X_ ^a^		
T	DLG‐σ_X_ interaction	DLG‐σ_X_ interaction^b^			
DLG‐σ_Y_ interaction^a^					

T, transmission.

^a^ Significant for two of the three beam models.

^b^ Significant for one of the beam models.

The influence of a given parameter can be quantified by the maximum amplitude induced by a change of level of the parameter on the output factors. For example, all significant parameters for field sizes larger or equal to 1 cm x 1 cm had a maximum amplitude of 2.3% meaning that tuning these parameters over the range studied could affect the calculated output factor of up to 2.3%. For some parameters, like the transmission for the 3 cm x 3 cm and 4 cm x 4 cm fields, the amplitude was very small (<0.2%) although significant. For comparison, the amplitude of σ_Y_ for the 0.5 cm x 0.5 cm field size could be up to 14%.

**Fig. 3:**
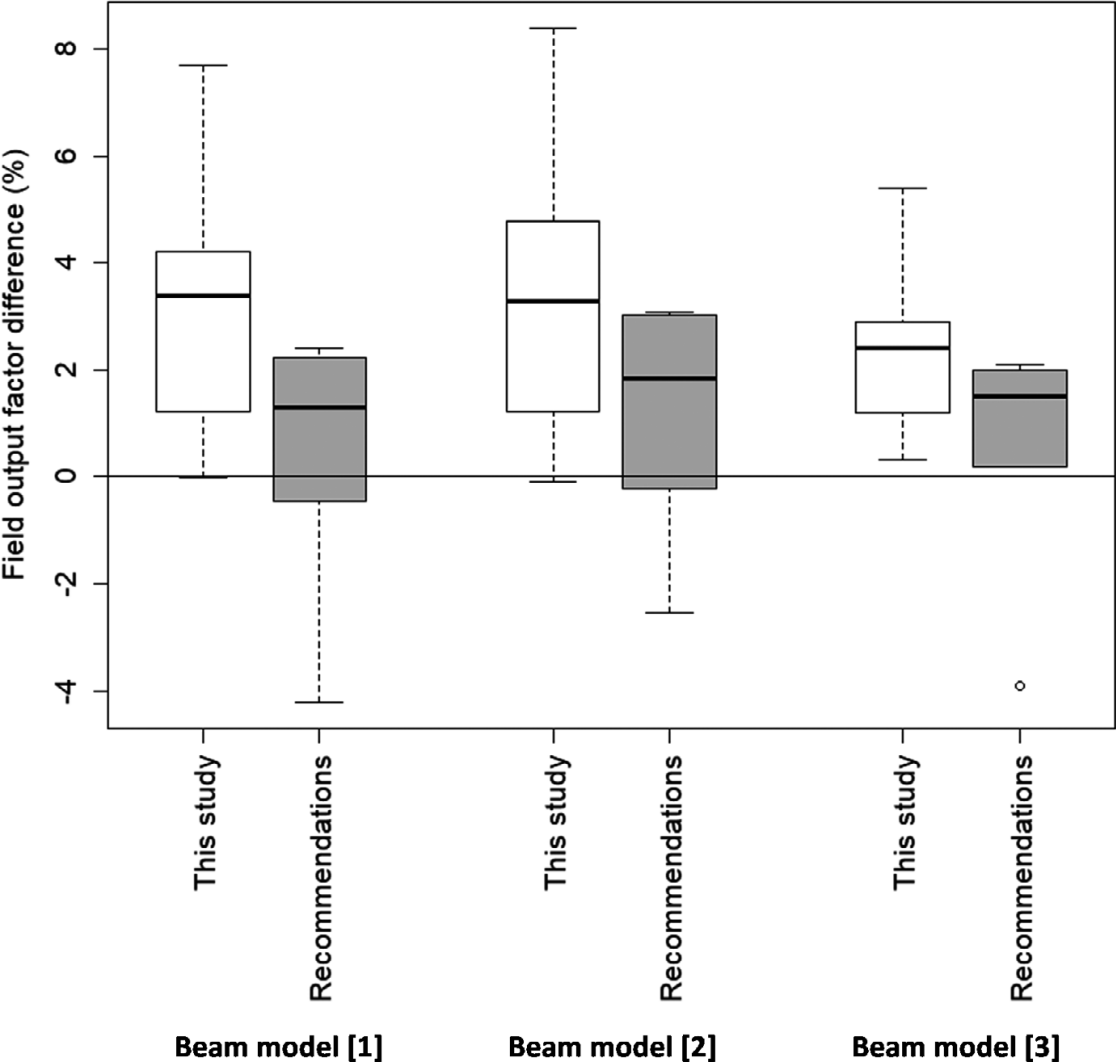
Deviation between calculated and measured field output factors using either the parameters found in this study or the manufacturer’s recommendations. Beam model [1]: TrueBeam Tx ‐ Millennium MLC ‐ X6; Beam model [2]: TrueBeam STx ‐ 120 HD MLC ‐ X6; Beam model [3]: TrueBeam STx ‐ 120 HD MLC ‐ X6 FFF.

Figure 3 represents the deviations between measured and calculated small field output factors. Two methodologies were followed to calculate the field output factors: one using the beam parameters optimizing the penumbra and the aOSG tests (given in Table 2) and one following the manufacturer recommendations. The median deviations were larger for the parameters found in this study but the differences were found not significant (paired t‐test performed on each beam studied, α = 5%)

### 3.4 Patient quality controls

Results of the patient quality controls performed with EBT3 films are given in Table 4. The mean gamma pass rate exceeds the universal tolerance limit of 95% given by the AAPM Task Group No. 218^18^ and all plans passed the universal action limit of 90%. Following the manufacturer’s recommendations, similar gamma passing rates were found. Differences between the two beam models were not statistically significant for a 3% ‐ 2 mm gamma analysis as well as a 3% ‐ 1 mm gamma analysis (paired t‐test, α = 5%).

## 4 DISCUSSION

This study presents a robust methodology to determine the four parameters required by the AcurosXB beam model. It was tested for two MLCs and two energies.

The effective target spot size was adjusted by comparing calculated and measured penumbras. The parameter values that minimize the deviation are given in Table 2 and differ largely from the values recommended by the manufacturer: σ_X_ = 1.5 mm and σ_Y_ = 0 mm. However, using σ_X_ = 1.5 mm would generate a mean deviation of up to 1 mm between the calculated and measured penumbras whereas using σ_Y_ =0 mm would generate a smaller 0.2 mm mean deviation. According to Glenn et al.^10^ the most commonly used value for AcurosXB is 1 mm for σ_X_ and σ_Y_, no matter the energy or the MLC. This can be explained because the suggested value by Varian changed between version 11 and 13 from 1 to 1.5 mm for σ_X_ and from 1 to 0 mm for σ_Y_. The work of Fogliata et al.^2^ also suggested effective target spot size values of 1 mm in both directions. Differences observed with this study could be explained by the different version of AcurosXB and by the detector used for profile measurements as well as its orientation. The study of Gardner et al.^3^ concluded that a 0.75 mm effective target spot in both directions optimized the results of patient quality controls for a TrueBeam STx at 6 FFF equipped with a 120 HD MLC. It was considered in this study that the DLG or the transmission did not affect the penumbra adjustment and that σ_X_ (respectively σ_Y_) did not affect the crossline (respectively inline) penumbra adjustment. This point was confirmed by keeping σ_X_ (or σ_Y_) unchanged while varying the DLG, the transmission and σ_Y_ (or σ_X_). A maximum deviation of 0.1 mm on the calculated penumbra was found.

The DLG was adjusted by minimizing the mean dose deviation for aOSG tests. Corresponding DLG values for the three beams considered are given in Table 2 and can largely differ from the values measured with the sweeping gap method with for example a difference of 1.75 mm for the Millennium MLC. The optimal DLG was determined over 15 aOSG tests associated with different gaps and TG fractions in order to encompass a maximum of clinical situation and have a beam model adjusted for all treatments. However, selecting specific gaps or TG fractions can modify the optimized DLG as shown by Hernandez et al.^19^ and such selection could be necessary if a beam model was to be used for a specific treatment like SRS brain for example as was performed by Gardner et al.^3^ Viellevigne et al.^6^ for example decided to select aOSG tests for TGi values representative of their clinical plans (0.2–0.5). Several studies adjusted the DLG parameter by optimizing patient quality controls and concluded that the DLG measured with the sweeping gap tests had to be increased between 0.4 and 1.7 mm for a 120 HD MLC.^6^, ^7^, ^8^, ^9^ This result is in agreement with this study although the methodology to determine the optimal DLG differed. It was considered in this study that the effective target spot size did not affect the aOSG tests. This point was confirmed by keeping the DLG unchanged while varying the effective target spot size. No difference on the calculated dose was found for the aOSG tests confirming results from Hernandez et al.^19^ The transmission was found to affect the calculated dose of the aOSG tests and one could imagine an adjustment of the transmission based on the aOSG tests. This choice was not made because transmission can be explicitly measured with a minimal variation (<0.3%) with the measurement conditions^20^ which is not the case for the DLG as previously stated.

**Fig. 4:**
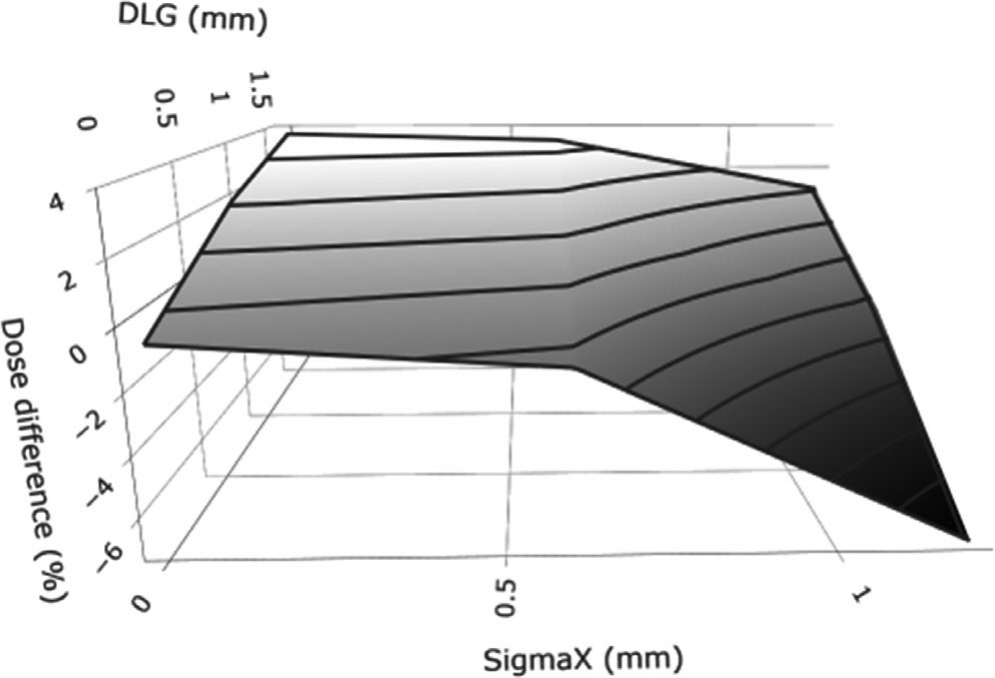
Representation of the DLG‐σX interaction for the 0.5 cm x 0.5 cm field output factor of the TrueBeam STx ‐ 120 HD MLC ‐ X6 FFF beam model.

The design of experiment methodology revealed that the parameters influencing the small field output factors depended on the field size. The amplitude of the parameters was large for the smallest field size but quite small (<3%) for the other field sizes. Below 2 cm x 2 cm, significant interactions also have to be taken into account. A significant interaction implies that the influence of a parameter depends on the value assigned to the other parameter and can thus not be investigated individually. An example of a significant interaction is illustrated in Fig. 4: although the variation of the dose difference as a function of DLG and σ_X_ is between −1% and 3% for most of the domain studied, there is a combination of the parameters (DLG = 0 mm and σ_X_ = 1.2 mm) for which the dose difference drops to −7%.

The originality of this study resides in highlighting that the choice of the optimal parameters (effective target spot size, DLG, and transmission) that maximizes the agreement between the calculated and measured small output factors must be done by considering the interaction between DLG and effective target spot sizes for field sizes smaller than 2 cm x 2 cm. When considering the values of the parameters described in Table 2, a maximum deviation of 8.4% was found between measured and calculated output factors, in line with Fogliata et al.^13^ This maximum deviation was reduced to 4.2% when considering the manufacturer recommendations but the mean deviation between the two methodologies was not significant. Moreover, other values of the beam parameters could probably give a better agreement between measured and calculated output factors but at the cost of larger differences on the penumbras. The methodology described here favored an agreement between calculated and measured penumbras over a decrease of the deviation between calculated and measured small output factors.

All patient quality controls realized with EBT3 films passed the universal action limit of 90% validating the custom parameters chosen for the beam models. Neither the gain in penumbra accuracy nor the loss in small field output factors agreement could be observed when comparing to the methodology provided by the manufacturer. A possible explanation could be the small magnitude of the differences expected (maximum 1 mm for the penumbra, maximum 1.5% for field output factors larger or equal to 1 cm) which cannot be detected with a 3% ‐ 2 mm or a 3% ‐ 1 mm gamma analysis. A bias in the comparison of the two methodologies was also introduced because plans were reoptimized in order to provide a clinically acceptable plan when using either the parameters found in this study or the manufacturer recommendations.

## 5 CONCLUSION

This study presented and validated in three beam models a new methodology to determine the effective target spot size and DLG: tuning the effective target spot size in order to minimize the difference between calculated and measured penumbras and tuning the DLG by minimizing the mean dose deviation for aOSG tests in order to take into account the tongue‐and‐grove effect. It was shown with a design of experiments methodology that tuning the parameters in order to minimize the difference between calculated and measured small field output factors had to be done with caution since all parameters and many interactions can influence the calculated output factors. As a consequence, we recommend prioritizing adjusting the effective spot sizes based on field penumbras.

## Conflict of interest

The author have no relevant conflicts of interest to disclose.

## Author Contribution

S. Dufreneix, M. Barreau, and T. Tiplica were responsible for the study design. V. Passal was responsible for the data acquisition, analysis, and interpretation. V. Passal and S. Dufreneix drafted the article. All authors read and approved the final manuscript.

## Supporting information


**Table S1**. Description of the 27 tests associated with the Taguchi L27 design of experiment. Each parameter can take three levels (1, 2, or 3).
