# Supplementary material for: Optimizing the effective spot size and the dosimetric leaf gap of the AcurosXB algorithm for VMAT treatment planning
Source: J Appl Clin Med Phys. 2021 May 27;22(6):154–61. doi: 10.1002/acm2.13256 (PMC8200512; doi:10.1002/acm2.13256)
Supplement: Supplementary file 1 — Table S1. Description of the 27 tests associated with the Taguchi L27 design of experiment. Each parameter can take three levels (1, 2, or 3). [file ACM2-22-154-s001.docx]

**Supplementary data:**

Description of the 27 tests associated with the Taguchi L27 design of experiment. Each parameter can take three levels (1, 2 or 3).

| Test | Parameter 1 σ_X_ | Parameter 2 σ_Y_ | Parameter 3 DLG | Parameter 4 T |
| --- | --- | --- | --- | --- |
| 1 | 1 | 1 | 1 | 1 |
| 2 | 1 | 1 | 2 | 2 |
| 3 | 1 | 1 | 3 | 3 |
| 4 | 1 | 2 | 1 | 2 |
| 5 | 1 | 2 | 2 | 3 |
| 6 | 1 | 2 | 3 | 1 |
| 7 | 1 | 3 | 1 | 3 |
| 8 | 1 | 3 | 2 | 1 |
| 9 | 1 | 3 | 3 | 2 |
| 10 | 2 | 1 | 1 | 2 |
| 11 | 2 | 1 | 2 | 3 |
| 12 | 2 | 1 | 3 | 1 |
| 13 | 2 | 2 | 1 | 3 |
| 14 | 2 | 2 | 2 | 1 |
| 15 | 2 | 2 | 3 | 2 |
| 16 | 2 | 3 | 1 | 1 |
| 17 | 2 | 3 | 2 | 2 |
| 18 | 2 | 3 | 3 | 3 |
| 19 | 3 | 1 | 1 | 3 |
| 20 | 3 | 1 | 2 | 1 |
| 21 | 3 | 1 | 3 | 2 |
| 22 | 3 | 2 | 1 | 1 |
| 23 | 3 | 2 | 2 | 2 |
| 24 | 3 | 2 | 3 | 3 |
| 25 | 3 | 3 | 1 | 2 |
| 26 | 3 | 3 | 2 | 3 |
| 27 | 3 | 3 | 3 | 1 |
